# Complete genome sequences of *Rhizobium favelukesii* strains T136, T1470, and T1473 from Canada

**DOI:** 10.1128/mra.01239-25

**Published:** 2026-02-09

**Authors:** Eden S. P. Bromfield, Sylvie Cloutier

**Affiliations:** 1 Agriculture and Agri-Food Canada, Ottawa, Canada; University of Strathclyde, Glasgow, United Kingdom

**Keywords:** *Rhizobium favelukesii* strains T136, T1470, and T1473, type III secretion system genes, nodulation and nitrogen fixation genes, complete genome sequences, *Medicago sativa*, *Melilotus albus*, Canada

## Abstract

We report complete genome sequences of *Rhizobium favelukesii* strains T136, T1470, and T1473 isolated from root-nodules of *Medicago* and *Melilotus*. Each genome comprises a chromosome (~4.2 Mb) and four to seven plasmids (~0.01–1.99 Mb) and contains predicted nodulation and nitrogen-fixation genes but lacks predicted type III secretion system genes.

## ANNOUNCEMENT

*Medicago sativa* (alfalfa) and *Melilotus albus* (white sweet clover) are agronomically important legumes that form effective nitrogen-fixing symbioses with *Sinorhizobium meliloti* and *S. medicae* (1). However, the acid-tolerant species *Rhizobium favelukesii* (2) predominates in certain soils and fixes nitrogen suboptimally with these hosts and with *Phaseolus vulgaris* (2–4). Previously, bacterial strains were isolated in 1992 from surface-sterilized nodules of *M. sativa* and *M. albus* grown at a Canadian field site (45.3851° N, 75.7043° W) (5) and were later assigned to *R. favelukesii* based on housekeeping gene sequence analysis (3).

Three of these strains (T136, T1470, and T1473) stored at Agriculture and Agri-Food Canada in 20% glycerol at −80°C were grown on modified tryptone-yeast-extract (TY) agar (6) for 3 days at 28°C. Genomic DNA was extracted using the Wizard SV Genomic DNA Purification System (Promega), purified with the DNeasy PowerClean Pro Kit (Qiagen), and sheared using Covaris g-TUBEs without size selection. Libraries prepared with the SMRTbell Express Template Prep Kit 2.0 (PacBio) were sequenced on a PacBio Sequel II platform (CLR chemistry) at Genome Québec (Canada). Raw data were filtered using SMRT Link v10.1. Sequencing metrics, including total raw polymerase reads, assembly coverage, filtered subreads, and subread N50 values, are summarized in Table 1. *De novo* assembly and circularization were performed using Flye v2.9 (7) (strains T136, T1473) and SMRT Link v10.1 (strain T1470) with default parameters. Circularity was confirmed by identifying and automatically trimming overlapping contig ends without additional rotation. Assembly quality was assessed via CheckM v1.2.4 using the *Rhizobium* marker set (8). Genomes were annotated with NCBI PGAP v5.3 (T136) and v6.7 (T1470, T1473) (9).

**TABLE 1:** Characteristics^*a*^ of genome sequences of *Rhizobium favelukesii* strains T136, T1470, and T1473 relative to reference strain *R. faveluskii* LPU 83^T^

Characteristic		T136	T1470	T1473	LPU83^T^ (reference)
Sequencing metrics					
Total raw (polymerase) reads; assembly coverage, ×		2,406,805; 2,176×	4,331,074; 3,342×	3,783,081; 2,871×	na
Filtered subreads (number; mean, bp; N50, bp)		832,792; 7,750; 10,465	4,331,074; 7,338; 10,212	3,783,081; 7,365; 10,218	na
Genome characteristics					
Genome size, bp (no. contigs)		7,738,336 (5)	8,104,678 (8)	8,004,949 (6)	7,569,648 (63)
Replicon size, bp (accession no.)	Chromosome	4,176,994 (CP088010)	4,230,318 (CP148091)	4,230,224 (CP149873)	4,195,305 (HG916852)
	Plasmid a	9,644 (CP088012)	20,514 (CP148098)	125,962 (CP149875)	151,687 (HG916853)
	Plasmid b	757,934 (CP08813)	28,606 (CP148097)	173,246 (CP149876)^*b*^	530,839 (**CBYB01**) (58 contigs)
	Plasmid c	806,677 (**CP088011**)	143,020 (CP148096)	802,278 (CP149877)	759,787 (HG916854)
	Plasmid d	1,987,087 (CP088009)	206,332 (CP148095)^*b*^	910,625 (**CP149874**)	1,932,030 (HG916855)
	Plasmid e	na	802,663 (CP148094)	1,762,614 (CP149878)	na
	Plasmid f	na	910,611 (**CP148093**)	na	na
	Plasmid g	na	1,762,614 (CP148092)	na	na
GC content (%)		59.5	59.5	59.5	59.5
Annotation features					
Total predicted genes		7,614	8,040	7,970	7,696
Total predicted protein-coding genes (CDSs)		6,833	7,127	7,064	7,617
Predicted rRNAs (5S, 23S, 16S)		3, 3, 3	3, 3, 3	3, 3, 3	3, 3, 3
Predicted tRNAs		51	52	52	51
Predicted nodulation genes		*nodD1D2D3ABCQIDEFGHJPLG*; *noeAB*; *nolFGN*	*nodD1D2D3ABCQIDEFGHJPLG*; *noeAB*; *nolFGN*	*nodD1D2D3ABCQIDEFGHJPLG*; *noeAB*; *nolFGN*	*nodD1D2D3ABCQIDEFGHJPLG*; *noeAB*; *nolFGN*
Predicted nitrogen fixation genes		*nifHDKENBQSUXAWZT*; *fixABCX*; *fixNOQP*; *fixGHIS*; *fixKLJU*; *fdxNB*; *rpoN*; *modABC*	*nifHDKENBQSUXAWZT*; *fixABCX*; *fixNOQP*; *fixGHIS*; *fixKLJU*; *fdxNB*; *rpoN*; *modABC*	*nifHDKENBQSUXAWZT*; *fixABCX*; *fixNOQP*; *fixGHIS*; *fixKLJU*; *fdxNB*; *rpoN*; *modABC*	*nifHDKENBQSUXAWZT*; *fixABCX*; *fixNOQP*; *fixGHIS*; *fixKLJU*; *fdxNB*; *rpoN*; *modABC*
Predicted type IV secretion system (T4SS) genes		*TraG/VirD4*; *TrbBCDEFGIJL*; *VirB1****-****6, 8-11*	*TraG/VirD4*; *TrbBCDEFGIJL*; *VirB1-6, 8-11*	*TraG/VirD4*; *TrbBCDEFGIJL*; *VirB1-6, 8-11*	*TraG/VirD4*; *TrbBCDEFGIJL*; *VirB1-6, 8-11*
Predicted type VI secretion system (T6SS) genes		nd	*tssABCDEFGHIJKLM*	nd	nd
Predicted hydrogen uptake genes		nd	*hupELSUV*; *hypABFCDE*; *nikEDCBAR*; *rpoN*	*hupELSUV*; *hypABFCDE*; *nikEDCBAR*; *rpoN*	nd
Predicted insertion sequences		302	546	546	184

^*a*^ Symbiosis plasmids are shown in bold type. Raw reads are total PacBio Sequel II polymerase reads; coverage (×) is based on total raw bases divided by genome size. Filtered subreads are adapter and quality trimmed reads used for assembly; mean and N50 were calculated from filtered subreads. na, not applicable; nd, not detected.

^*b*^ Uncircularized plasmid consisting of a single linear contig.

**Fig 1:**
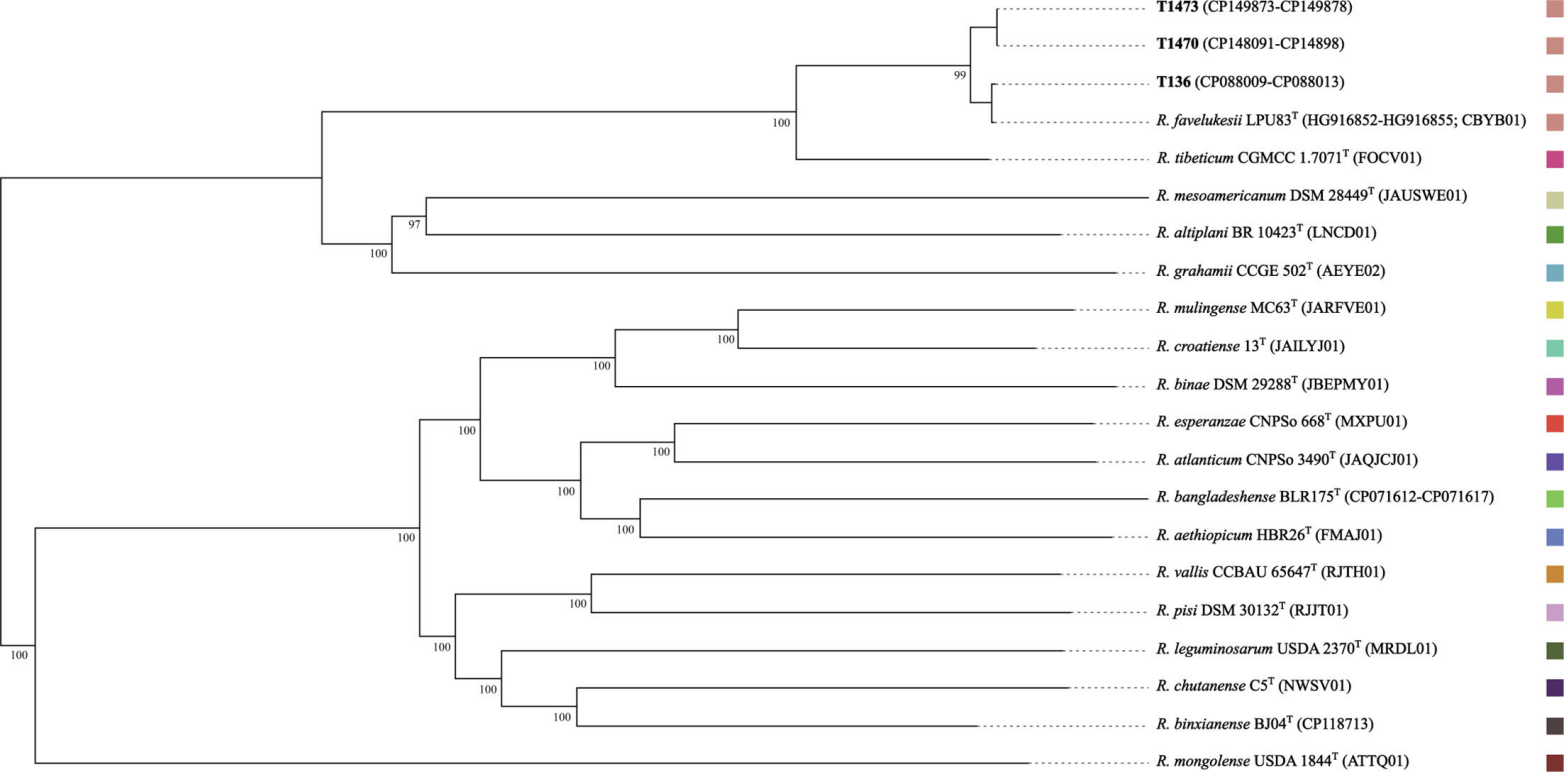
Phylogenomic tree inferred using FastME (v.2.1.6.1) from Genome BLAST Distance Phylogeny (GBDP) distances generated through the TYGS pipeline (12), showing the placement of *Rhizobium favelukesii* strains T136, T1470, and T1473 relative to reference taxa of the genus *Rhizobium*. Branch lengths are scaled according to the GBDP distance formula d5. Numbers above branches represent GBDP pseudo-bootstrap support values based on 100 replications. Solid colors denote TYGS species assignments; strains sharing the same color are assigned to the same species. Sequence accession numbers are provided in brackets.

FastANI v1.34 (10) values for T136, T1470, and T1473 versus LPU83ᵀ (2, 11) were 99.88, 99.17, and 99.18%, respectively, confirming their assignment to *R. favelukesii*; species affiliation was corroborated by their placement in a whole genome-based TYGS v403 tree inferred using FastME (v 2.1.6.1) from Genome BLAST Distance Phylogeny distances (12) (Fig. 1).

Table 1 shows genomic characteristics of novel strains compared with *R. favelukesii* reference strain LPU83^T^ (11). The genome of each novel strain comprises a chromosome (~4.2 Mb) and between four and seven plasmids (~0.01 to 1.99 Mb); plasmids were identified by the presence of predicted *repABC* genes (13). All replicons were circularized, except for pT1473b and pT1470d, each consisting of a single linear contig.

A symbiosis plasmid was identified in each strain, containing predicted genes for nodulation and nitrogen fixation, but predicted type III secretion system (T3SS) genes usually required for nodulation (14) were not detected.

Predicted conjugative type IV secretion system (T4SS) genes (15) were detected in all strains, whereas predicted type VI secretion system (T6SS) genes implicated in interbacterial competition (16), were present only in T1470. Predicted genes involved in hydrogen recycling from nitrogen fixation (17) were identified exclusively in T1470 and T1473. Numerous insertion sequences (ISs) predicted with ISEScan v1.7.3 (18) were present in all strains, indicating high genome plasticity and environmental adaptability (19).

These data represent a valuable resource for further genomic and comparative studies of *R. favelukesii*.

## Data Availability

The whole genome shotgun projects for *R. favelukesii* strains T136, T1470, and T1473 were deposited at DDBJ/ENA/GenBank as Assembly accession numbers GCA_021044665.1, GCA_042140505.1, and GCA_017571635.3, respectively. Raw PacBio data were deposited in the NCBI Sequence Read Archive as SRX25560886, SRX25736254, and SRX25674803 in BioProject accession numbers PRJNA781622, PRJNA1088506, and PRJNA715167, respectively. *R. favelukesii* strains T136, T1470, and T1473 were deposited in the BCCM/LMG Bacteria Collection, University of Ghent, Belgium, under the culture collection numbers LMG 32796, LMG 32376, and LMG 32377, respectively.
